# Time course of exercise induced alterations in daily activity in chronic fatigue syndrome

**DOI:** 10.1186/1476-5918-4-10

**Published:** 2005-10-28

**Authors:** Christopher D Black, Kevin K McCully

**Affiliations:** 1 Department of Kinesiology, The University of Georgia, Athens, GA, USA

## Abstract

In a previous study we demonstrated that while people with CFS had lower daily activity levels than control subjects, they were able to increase daily activity via a daily walking program. We reanalyzed our data to determine the time course of activity changes during the walking program. Daily activity assessed via an accelometer worn at the hip was divided into sleep, active, and walking periods. Over the first 4–10 days of walking the subjects with CFS were able to reach the prescribed activity goals each day. After this time, walking and total activity counts decreased. Sedentary controls subjects were able to maintain their daily walking and total activity goals throughout the 4 weeks. Unlike our previous interpretation of the data, we feel this new analysis suggests that CFS patients may develop exercise intolerance as demonstrated by reduced total activity after 4–10 days. The inability to sustain target activity levels, associated with pronounced worsening of symptomology, suggests the subjects with CFS had reached their activity limit.

We have previously published data suggesting that individuals with chronic fatigue syndrome (CFS) could increase their total daily physical activity over a period of four weeks [1]. Six individuals with CFS were prescribed a daily walking program ranging from 15–25 minutes per day with the hopes of increasing their daily activity to a level approximating that of a healthy sedentary person. Daily activity was measured by an accelometer worn at the waist [1]. We found that while our CFS subjects were able to increase their daily activity, they were unable to reach daily levels similar to sedentary controls. Unlike previous studies, our daily exercise program was accompanied by a worsening of CFS symptomology. Overall mood, daily fatigue, and time spent each day with fatigue all worsened over the course of four weeks as the exercise program progressed. Based upon the observation that our most "active" CFS subjects were the least able to increase their daily activity, we proposed a "daily activity limit" as a possible explanation for the worsening of fatigue related symptoms and the inability to reach activity levels of sedentary controls.

In order to further examine the idea of a "daily activity limit" a detailed analysis of each subject's activity each day was performed. Daily activity was broken into 3 categories – exercise, active, and sleep based upon activity per minute and time of day. Four of the six subjects appeared to perform a single bout of exercise each day, and were chosen for further analysis. When daily activity data was viewed in this manner, a distinct pattern was observed. During the first 4–10 (average of 7) days of exercise, our CFS subjects spent, on average, approximately 23 minutes each day exercising. This indicates that the subjects were not only complying with the prescribed exercise, but also were able to reach the daily exercise target. This resulted in total daily activity of the CFS patients being approximately equal to the baseline activity of the control sedentary subjects (Figure 1). However, over the final 3 weeks of prescribed exercise, the average time spent each day exercising fell to approximately 8 minutes per day. Interestingly, average time and counts per day spent sleeping, and during non-exercise "active" periods did not change over the course of the study. In contrast, sedentary control subjects responded to a similar daily walking program by increasing their total daily activity 25–30% for the first week, and were subsequently able to maintain this increase for three weeks additional weeks.

**Figure 1:**
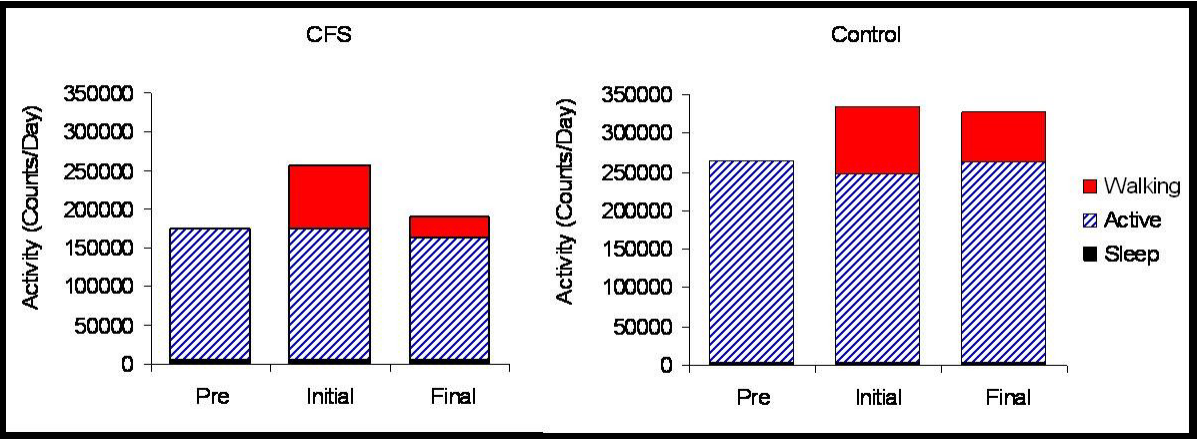
The figure shows average data for 4 CFS and 4 control subjects. Accelorometer counts at the waist were recorded every two minutes continuously for 6 weeks (2 weeks of Pre with no walking, 4–10 days of initial walking, and ~3 weeks of continued walking-Final). Activity is in counts per day.

In light of these new findings, we feel a new interpretation of our data is warranted. Unlike our initial interpretation that CFS subjects could maintain an activity increase over four weeks, it is now apparent that the CFS subjects were only able to sustain the prescribed increase in daily activity for 4–10 days. We believe the reduction in total daily activity levels, primarily from a reduction in time spent exercising, observed during the following 3 weeks were related to greater symptoms of fatigue. These results indicate that the CFS patients in the current study were more likely than controls to develop exercise intolerance. This conclusion is supported by a previous case report, and is often suggested in review articles [2,3]. Our results also provide information on the time course by which people with CFS may develop exercise intolerance. Whether CFS patients can train to increase their exercise tolerance is currently unknown.

## References

[B1] Black CD, O'Connor P J, McCully KK (2005). Increased daily physical activity and fatigue symptoms in chronic fatigue syndrome. Dyn Med.

[B2] Snell CR VJMSSRPSGDWL, Jason L FPTR (2003). Exercise Therapy. Handbook of Chronic Fatigue Syndrome.

[B3] MacDonald KL, Osterholm MT, LeDell KH, White KE, Schenck CH, Chao CC, Persing DH, Johnson RC, Barker JM, Peterson PK (1996). A case-control study to assess possible triggers and cofactors in chronic fatigue syndrome. Am J Med.

